# Effects upon metabolic pathways and energy production by Sb(III) and As(III)/Sb(III)-oxidase gene *aioA* in *Agrobacterium tumefaciens* GW4

**DOI:** 10.1371/journal.pone.0172823

**Published:** 2017-02-27

**Authors:** Jingxin Li, Birong Yang, Manman Shi, Kai Yuan, Wei Guo, Mingshun Li, Gejiao Wang

**Affiliations:** State Key Laboratory of Agricultural Microbiology, Huazhong Agricultural University, Wuhan, P. R. China; Universite Paris-Sud, FRANCE

## Abstract

*Agrobacterium tumefaciens* GW4 is a heterotrophic arsenite [As(III)]/antimonite [Sb(III)]-oxidizing strain. The As(III) oxidase AioAB is responsible for As(III) oxidation in the periplasm and it is also involved in Sb(III) oxidation in *Agrobacterium tumefaciens* 5A. In addition, Sb(III) oxidase AnoA and cellular H_2_O_2_ are also responsible for Sb(III) oxidation in strain GW4. However, the deletion of *aioA* increased the Sb(III) oxidation efficiency in strain GW4. In the present study, we found that the cell mobility to Sb(III), ATP and NADH contents and heat release were also increased by Sb(III) and more significantly in the *aioA* mutant. Proteomics and transcriptional analyses showed that proteins/genes involved in Sb(III) oxidation and resistance, stress responses, carbon metabolism, cell mobility, phosphonate and phosphinate metabolism, and amino acid and nucleotide metabolism were induced by Sb(III) and were more significantly induced in the *aioA* mutant. The results suggested that Sb(III) oxidation may produce energy. In addition, without periplasmic AioAB, more Sb(III) would enter bacterial cells, however, the cytoplasmic AnoA and the oxidative stress response proteins were significantly up-regulated, which may contribute to the increased Sb(III) oxidation efficiency. Moreover, the carbon metabolism was also activated to generate more energy against Sb(III) stress. The generated energy may be used in Sb transportation, DNA repair, amino acid synthesis, and cell mobility, and may be released in the form of heat.

## Introduction

Antimony (Sb) is widely present in soil and aquatic systems as a result of natural processes and human activities [1, 2]. It can exist in multiple oxidation states, with the most common being antimonite [Sb(III)] and antimonate [Sb(V)] [1]. Due to its affinity for the thiol groups of glutathione and proteins, Sb and its compounds are considered as priority pollutants by the United States Environmental Protection Agency [3] and the European Union [4]. The biogeochemical cycle of this element strongly depends on microbial transformation that affects the toxicity and mobility of antimony species in the environment [5, 6]. To thrive in Sb-rich environments, microbes have coped with the toxicity of Sb using various strategies [5]. Microbial Sb(III) oxidation, which transforms the toxic Sb(III) to the much less toxic Sb(V) could be used as a strategy for biochemical detoxification and considered a means of environmental Sb bioremediation.

Sb and arsenic (As) both belong to Group 15 in the Periodic Table and share some similar chemistries. Regarding the mechanisms of microbial Sb resistance, the *ars* operon conferring As(III) resistance is also responsible for Sb(III) resistance [7]. It is known that the As(III) efflux protein ArsB can bind with a dimer of ATPase ArsA to form an ATP-coupled efflux pump and catalyze the extrusion of As(III)/Sb(III) with hydrolysis of ATP [8, 9]. In addition, another trivalent metalloid/H^+^ antiporter Acr3p could also function as an Sb(III) efflux pump [10]. Microbial Sb(III) methylation and Sb(V) reduction appear to be widespread in the environment, although the genes and proteins involved in these processes have not been identified [11]. It has been found that microbial Sb(V) reduction was coupled to a dissimilatory respiratory pathway, which could conserve energy for bacterial growth [12, 13].

About 60 Sb(III)-oxidizing bacterial strains have been found and some of them can also oxidize arsenite [As(III)] to arsenate [As(V)] [14]. Recently, our group and collaborators demonstrated that the As(III) oxidase AioAB, which oxidizes the more toxic As(III) to the less toxic As(V) in the periplasm, could also catalyze Sb(III) oxidation in *Agrobacterium tumefaciens* 5A [15]. However, the deletion of *aioA* caused a null As(III) oxidation, but only decreased the Sb(III) oxidation efficiency by ~25% [15]. Subsequently, we found a cytoplasmic Sb(III) oxidase AnoA responsible for Sb(III) oxidation in *A*. *tumefaciens* GW4 [16]. Both *A*. *tumefaciens* GW4 and *A*. *tumefaciens* 5A are heterotrophic As(III)/Sb(III)-oxidizing bacteria, however, the As(III)/Sb(III) oxidation efficiency and resistance of strain GW4 are much higher than those of strain 5A [15–18]. Recently, we found that in contrast to strain 5A, the deletion of *aioA* increased Sb(III) oxidation efficiency in strain GW4, and the cellular H_2_O_2_ may act as a non-enzymatic factor for bacterial Sb(III) oxidation [19].

So far, only two chemoautotrophic bacteria, *Stibiobacter senarmontii* and *Variovorax paradoxus* IDSBO-4, have been found to produce energy for bacterial growth with the fixation of CO_2_ using Sb(III) as an electron donor [20, 21]. Previously, we showed that the heterotrophic strain GW4 could produce energy for bacterial growth from As(III) oxidation [22]. However, the heterotrophic Sb(III)-oxidizing bacteria that have been described were not shown to produce energy for growth from Sb(III) oxidation. In our previous study, global analysis of cellular responses to Sb(III) was performed using comparative proteomics with or without the addition of 50 μM Sb(III) in strain GW4 [16]. It was shown that Ars-resistance, Sb(III) oxidase AnoA, phosphate metabolism, carbohydrate metabolism, and energy generation were induced by Sb(III) [14, 16].

In the present study, we found that besides the increased Sb(III) oxidation efficiency, deletion of *aioA* also increased energy production, bacterial mobility and heat release, suggesting that *aioA* might affect the other metabolic pathways in response to Sb(III). To further investigate the energy metabolism driven by Sb(III) in strain GW4 and the effect of *aioA* on the global response to Sb(III), proteomics and transcriptional analyses were performed.

## Materials and methods

### Constructions of the Δ*aioA* mutant and complemented strains and detection of Sb(III) oxidation

The construction of an in-frame deletion strain GW4-Δ*aioA* and its complementary strain GW4-Δ*aioA-*C were performed in a previous work [22]. *A*. *tumefaciens* strains were grown in a chemically defined medium (CDM) [23] containing 0 or 50 μM K_2_Sb_2_(C_4_H_2_O_6_)_2_ [Sb(III)] and incubated at 28°C with 100 rpm shaking. The carbon source in the CDM medium is sodium lactate. When needed, 50 μM K_2_H_2_Sb_2_O_7_ [Sb(V)] was added to the CDM agar plate. Sb(III) oxidation was detected using HPLC-HG-AFS (Beijing Titan Instruments Co., Ltd., China) according to Li, et al. [24].

### Analysis of ATP and NADH contents

To test the ATP and NADH contents, *A*. *tumefaciens* strains were each inoculated into 100 mL of CDM medium. When OD_600_ reached 0.5–0.6, 0 or 50 μM Sb(III) was added to each culture. After incubation of 2 h, bacterial cells were harvested by centrifugation (13,400×g, 5 min, 4°C) and resuspended in 1 mL of 0.4 M perchloric acid with 1.0 mM EDTA. Then, the suspension was sonicated on ice for 5 min and centrifuged (13,400×g, 5 min, 4°C) to remove the debris. The pH of the supernatant was adjusted to 7.0 with 1.0 M K_2_CO_3_, and the samples were subsequently filtered (0.22 μm filter) for HPLC analysis (HPLC 2690 series, Waters, Massachusetts, USA). At the same time, samples were taken for detecting the protein concentration using Coomassie Brilliant Blue method [25]. ATP and NADH in each sample were identified by comparing the retention times to standards, and the peak area represented the content of each energy substance. The mobile phase contained 90% 50 mM phosphate buffer, 10% acetonitrile and 3.22 g/L tetrabutylammonium bromide (pH 6.8). The flow velocity of the mobile phase was 1 mL/min. To eliminate error, each text was replicated three times.

### Mobility assay

*A*. *tumefaciens* strains were each inoculated into 5 mL of CDM medium and incubated at 28°C with 120 rpm shaking. When OD_600_ reached 0.1–0.2, the strains were each deposited at the center of the soft agar plate (CDM with 0.25% agar) with the addition of 0, 10, 25, 50, 200, 500 μM Sb(III) or Sb(V). Then, the plates were incubated at 28°C for 72 h, and the swim ring diameters were measured to quantify the extent of mobility [26].

### Microcalorimetric measurement

Metabolic activities of *A*. *tumefaciens* strains were evaluated with a third generation thermal activity monitor (TAMIII, Sweden) [27]. Bacterial cells were each inoculated into 5 mL of CDM medium and incubated at 28°C with 120 rpm shaking. When OD_600_ reached 0.1–0.2, the strains were transferred into a sterilized steel ampoule, respectively. Meanwhile, 0 or 50 μM Sb(III) was added into each ampoule, which was then sealed with a cap and put into the TAM air isothermal microcalorimeter. The temperature of the calorimeter system and the isothermal box were controlled at 28°C. The growth process was monitored continuously and its thermogenic curve was obtained.

### Proteomic and genomic analyses

Comparative proteomics analyses were performed in strain GW4 with or without the addition of 50 μM Sb(III) [16] and in wild type GW4 and mutant strain GW4-Δ*aioA* in the presence of 50 μM Sb(III). Bacterial cells were cultivated in CDM medium at 28°C with 120 rpm shaking. Mid exponential phase cells (32 h) were harvested by centrifugation at 13,400×g for 10 min at 4°C. Bacterial cells were washed three times with ice-cold PBS buffer (pH 7.4) and resuspended in 0.5 mL of lysis buffer (7 M urea, 2 M thiourea and 4% w/v CHAPS) with the addition of 1% (w/v) DTT and protease inhibitor cocktail (Amerso, USA). Subsequently the cells were treated by ultrasonication for 5 min and incubated with the addition of 7.5 μM/mL DNase I and 10 μM/mL RNaseA at 28°C for 1 h. Protein extracts were obtained by centrifugation (13,400×g, 10 min, at 4°C) to remove cell debris and the concentration was determined with the Coomassie Brilliant Blue method using BSA as a standard. A volume equivalent to 500 μg of protein extracts was purified using the ReadyPrep 2-D Clean-up Kit (Bio-Rad Laboratories, Philadelphia, PA) and further used for two-dimensional gel electrophoresis.

Purified protein samples (100 μg) were diluted with rehydration buffer (7 M urea, 2 M thiourea, 2% CHAPS, 65 mM DTT, 0.5% IPG buffer pH 4–7 and 0.002% bromophenol blue) to a total volume of 300 μL per immobiline dry strip and run on the isoelectric focusing (IEF) IPG-strip gels (pH 4–7, 18 cm, GE). The IPG gels were first loaded by passive rehydration for 4 h and then by active rehydration at 50 V for 12 h. After IEF, the IPG strips were first balanced in equilibration buffer I (6 M urea, 2% SDS, 50 mM Tris-HCl pH 8.8, 20% glycerol and 1% DTT) for 15 min and subsequently in equilibration buffer II (6 M urea, 2% SDS, 50 mM Tris-HCl pH 8.8, 20% glycerol and 4% iodacetamide) for another 15 min. The equilibrated strips were each loaded on the top of 12% SDS separating polyacrylamide gels, and covered with 0.5% (w/v) low melting point agarose prepared with the same SDS separation buffer but containing a trace amount of bromophenol blue. Electrophoresis was carried out using a Dodeca Cell system (Bio-Rad) at a constant current of 20 mA for 1 h and followed by 40 mA until the marker dye reached the bottom of the gels. Then, the protein spots in analytical gels were visualized using silver staining method, and recorded as digitalized images using a GS-800 calibrated densitometer (BioRad). Three replicate gels were performed for each sample.

The images were analyzed with PDQuest software (version 8.0, BioRad) and all of the gels in the MatchSet were normalized on the basis of the total quantity in valid spots. Proteins were considered differentially expressed when the average values exceeded the 2-fold threshold with a p-value <0.05. Protein spots of interest were manually extracted from gels as previously described [16] and analyzed by MALDI-TOF-MS (Bruker Daltonik GmbH, Bremen, Germany) [28]. Proteins were identified by peptide mass fingerprinting (PMF) through Bruker Ultraflex automated data analysis software and searched with a local MASCOT search engine (Matrix Science, London, UK).

The genomic analysis was conducted through blastn and blastp in the genome of *A*. *tumefaciens* GW4 on the NCBI website (http://www.ncbi.nlm.nih.gov). Metabolic pathway mapping was performed using the KEGG (http://www.genome.jp/kegg/) database [29, 30].

### Quantitative RT-PCR analysis

The *A*. *tumefaciens* strains were cultured as described above. Bacterial cells used for RNA isolation were collected at the same time as the protein extracted for proteomics. Total RNA was extracted using Trizol reagent (Invitrogen) and treated with RNase-free DNase I (Takara) at 37°C for 1 h to remove genomic DNA. Then the reaction was terminated by adding 50 mM EDTA at 80°C for 10 min. The quality and quantity of the RNA were determined with a spectrophotometer (NanoDrop 2000, Thermo). Reverse transcription was performed with a RevertAid First Strand cDNA Synthesis Kit (Thermo) with 300 ng of total RNA for each sample [31]. Then the obtained cDNA was diluted 10-fold for quantitative RT-PCR analysis by ABI VIIA7 in a 0.1 mL Fast Optical 96-well Reaction Plate (ABI) using SYBR^®^ Green Realtime PCR Master Mix (Toyobo). Three technical and biological replicates were established for each reaction with the primers listed in S1 Table. The cycle threshold (Ct) value was measured, and gene expression was normalized by ΔΔCT analysis with an iQ5 Real-Time PCR Detection System (Bio-Rad, USA).

## Results and discussion

### Deletion of the periplasmic As(III)/Sb(III) oxidase gene *aioA* (strain GW4-Δ*aioA*) increased Sb(III) oxidation efficiency against Sb(III) stress

The As(III) oxidase gene *aioAB* was responsible for periplasmic Sb(III) oxidation in *A*. *tumefaciens* 5A. To investigate the effect of *aioA* on Sb(III) oxidation in strain GW4, the *aioA* mutant strain (GW4-Δ*aioA*) and complemented strain (GW4-Δ*aioA-*C) were obtained from a previous study [22]. The large subunit encoding gene *aioA* is a single copy in the genome of *A*. *tumefaciens* GW4 (NCBI Accession No. AWGV00000000) [16]. Recently, we found that the deletion of *aioA* increased Sb(III) oxidation efficiency in strain GW4, and the cellular content of H_2_O_2_ was also increased in strain GW4-Δ*aioA*. The complemented strain GW4-Δ*aioA-*C showed similar Sb(III) oxidation efficiency and H_2_O_2_ content to those of the wild-type strain GW4 [19]. Thus, we predicted that the deletion of the periplasm As(III)/Sb(III) oxidase gene *aioA* may affect other metabolic pathways associated with the Sb(III) detoxification and oxidative stress response in strain GW4.

### The chemotaxic ability toward Sb(III) was increased in strain GW4-Δ*aioA*

**Fig 1:**
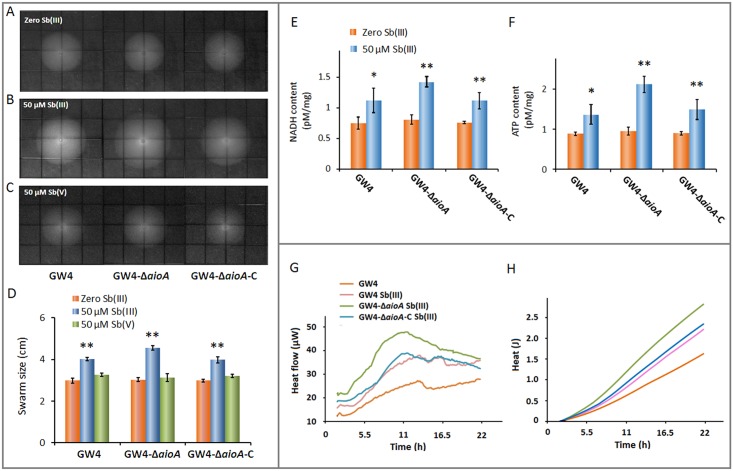
Chemotaxis response, energy generation and microcalorimetric analyses of *A*. *tumefaciens* strains. In swarm plate assay, bacterial cells were inoculated on CDM semisolid agar plates with or without the addition of 50 μM Sb(III) or Sb(V) and incubated at 28°C. (A) CDM semisolid agar plate (negative control). (B) Positive chemotaxis toward 50 μM Sb(III). (C) No chemotaxis toward 50 μM Sb(V). (D) Diameter of the swarming ring. Data are shown as the mean of three replicates, with the error bars representing ± SD. (E) and (F) The cellular contents of NADH and ATP in *A*. *tumefaciens* strains GW4, GW4-Δ*aioA* and GW4-Δ*aioA-*C, with or without the induction of 50 μM Sb(III). The contents of NADH and ATP were tested by HPLC. Data are shown as the mean of three replicates, with the error bars representing ± SD. ** represents p<0.01; *represents p<0.05. (G) and (H) Thermogenic and heat output curves of *A*. *tumefaciens* strains during growth at 28°C. Bacterial cells were each inoculated into 5 mL of CDM medium and incubated at 28°C with 120 rpm shaking. When OD_600_ reached 0.1–0.2, 0 or 50 μM Sb(III) was added to the culture for microcalorimetric measurement using a third generation thermal activity monitor (TAMIII, Sweden).

To investigate how *A*. *tumefaciens* strains respond to Sb in chemotaxis, swarm plate assays were conducted on semisolid agar plates with or without the addition of different concentrations of Sb(III) or Sb(V). As shown in Fig 1A and 1D, no significant difference in the swarming diameter was observed among the three *A*. *tumefaciens* strains without Sb(III). However, strain GW4 exhibited a positive chemotaxis and mobility in response to 50 μM Sb(III), and the deletion of *aioA* increased chemotaxis towards Sb(III) (Fig 1B and 1D). The complemented strain showed a similar swarming diameter to that of wild-type strain GW4 (Fig 1B and 1D). In the presence of 10 μM and 25 μM Sb(III), the swarming diameters showed no obvious difference compared with that of 50 μM Sb(III) (data not shown). However, with high amounts of Sb(III) (e.g. 200, 500 μM), the growth of GW4 was inhibited and the strain would swarm away from it due to the high toxicity of Sb(III) (data not shown). In addition, strains GW4, GW4-Δ*aioA*, and GW4-Δ*aioA*-C exhibited no chemotaxis towards Sb(V), suggesting that the positive chemotaxis towards Sb(III) was due to the energy generated from the process of Sb(III) oxidation, rather than bacterial chemotaxis towards Sb(V) (Fig 1C and 1D).

### The cellular contents of ATP and NADH were increased by Sb(III) in strain GW4-Δ*aioA*

To investigate the energy production affected by Sb(III) and *aioA*, the cellular contents of ATP and NADH were measured in strains GW4, GW4-Δ*aioA* and GW4-Δ*aioA-*C with or without the addition of 50 μM Sb(III). The contents of ATP and NADH in the three *A*. *tumefaciens* strains showed no significant difference without Sb(III) (Fig 1E and 1F). The contents of ATP and NADH were increased in strain GW4 (p<0.05) and further increased in strain GW4-Δ*aioA* (p<0.01) with the addition of Sb(III) (Fig 1E and 1F). As expected, the complemented strain GW4-Δ*aioA-*C recovered the phenotype back to the wild-type strain (Fig 1E and 1F). The results indicated that Sb(III) could induce the energy generation of *A*. *tumefaciens* strains and the large amount of ATP and NADH in strain GW4-Δ*aioA* in the presence of Sb(III) suggested that the *aioA* mutant strain might require more energy to tolerate the toxicity of Sb(III).

### Microcalorimetry analysis indicated that Sb(III) significantly increased heat production in strain GW4-Δ*aioA*

To further understand the energy utilization in *A*. *tumefaciens* strains, heat production was measured using microcalorimetry. The metabolic thermogenesis curves of *A*. *tumefaciens* strains can be divided into four phases: the lag phase, active recovery phase, stationary phase and decline phase (Fig 1G), which was consistent with those of other bacteria [32]. In addition, the peak power (*P*_max_) of strain GW4 was found to increase in the presence of Sb(III) and was highest in that of strain GW4-Δ*aioA* (Fig 1G). The phenotype of the complemented strain GW4-Δ*aioA*-C was recovered to the wild-type strain. The area under the curve is the heat output released by *A*. *tumefaciens* strains during the metabolic process. The heat output was induced by Sb(III) in strain GW4 and significantly increased in strain GW4-Δ*aioA* in the presence of Sb(III) (Fig 1H). The heat output curves demonstrated that Sb(III) increased the heat production in strain GW4, and more heat was produced in the *aioA* mutant to resist the toxicity of Sb(III).

### Comparative proteomic analysis of *A*. *tumefaciens* GW4 and GW4-Δ*aioA* with the addition of Sb(III)

**Fig 2:**
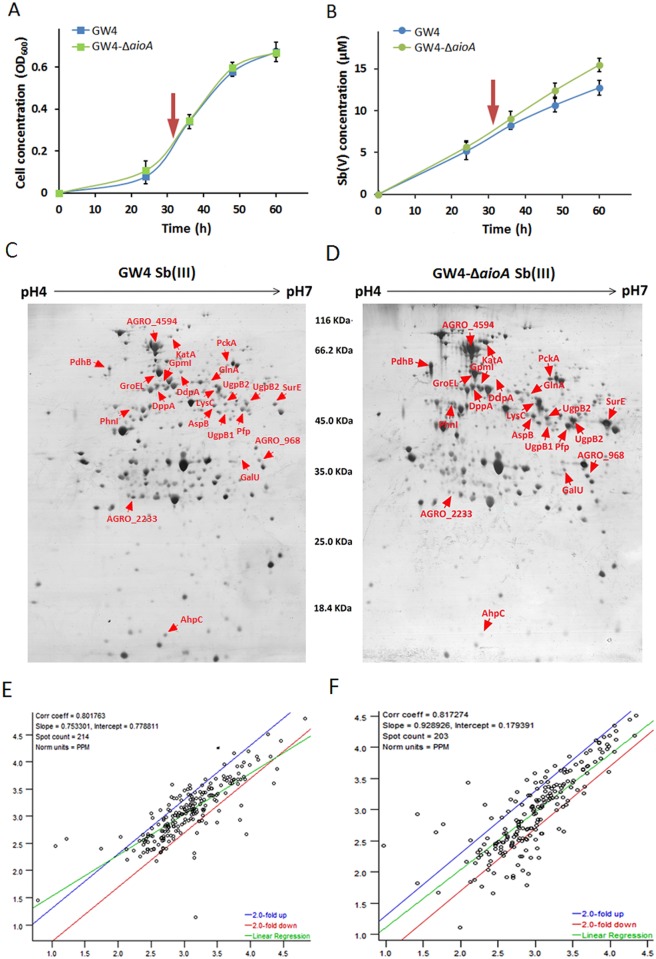
Growth, Sb(III) oxidation and proteomics of strains GW4 and GW4-Δ*aioA* with the addition of 50 μM Sb(III). (A) Growth profiles of strains GW4, GW4-Δ*aioA* in CDM medium containing 50 μM Sb(III). (B) Sb(III) oxidation curves of the same strains under the same culture conditions. The Sb(V) content was measured by HPLC-HG-AFS. The arrow indicates the sampling time for proteins used for 2-D electrophoresis detection (mid exponential phase). (C) and (D) The 2-D electrophoresis gels of total proteins in strain GW4 and GW4-Δ*aioA* grown with the addition of 50 μM Sb(III), respectively (an example from triplicate experiments). Arrow heads indicate proteins with differential expression. (E) and (F) Correlation coefficients of triplicate 2-D gels of strains GW4 and GW4-Δ*aioA*, respectively.

To investigate the effect of *aioA* on the global response to Sb(III) in *A*. *tumefaciens* GW4, comparative proteomic analysis of strains GW4 and GW4-Δ*aioA* was performed with the addition of 50 μM Sb(III). In a previous study, we analyzed bacterial responses to Sb(III) using comparative proteomics with or without the addition of 50 μM Sb(III) in wild-type strain GW4 [16]. To make the results comparable, the culture condition and time point for samples being taken were consistent with our previous study. Bacterial cells were inoculated into CDM medium supplemented with 50 μM Sb(III). After 32 h of incubation (at the midexponential phase), samples were taken for proteomic analysis. At this time, Sb(III) had no effect on the growth of strain GW4-Δ*aioA*, and the Sb(III) oxidation rate was slightly increased in contrast with the wild-type strain GW4 (Fig 2A and 2B), which is consistent with our previous study [19].

Proteomic analysis was carried out with IPG gradient gels covering pH 4–7 and 12% PAGE gels to obtain sufficient protein separation. An average of 214 and 203 protein spots were detected in the samples of strains GW4 and GW4-Δ*aioA*, respectively. Example 2-DE gel patterns are shown in Fig 2C and 2D. The correlation of triplicate gels of these two samples were 0.80 and 0.82, respectively (Fig 2E and 2F), indicating good reproducibility. Thirty stable spots exhibiting at least 2-fold change were chosen for MALDI-TOF-MS analysis, 21 of which were successfully identified by Peptide Mass Fingerprinting (PMF) (Table 1). Among them, 16 proteins were up-regulated and 5 proteins were down-regulated in the *aioA* mutant with Sb(III). Based on KEGG pathway assignments, they were distributed in stress responses, carbohydrate and energy metabolism, phosphonate and phosphinate metabolism, amino acid synthesis and metabolism, nucleotide metabolism, and unknown functions (Table 1).

**Table 1:** Information of the proteins/genes with different expression ratio in proteomics and qRT-PCR.

Gene name	Protein name	Accession No.	Expression ratio		
			GW4 -Sb/+Sb	GW4-Δ*aioA* -Sb/+Sb	GW4 +Sb/GW4-Δ*aioA* +Sb
**Antimony oxidation and resistance**					
*aioA*	Arsenite oxidase	KDR86840	1.2	0	0 ↓
*anoA*	Antimonite oxidase	KDR88348	4.1*↑	3.7 **↑**	2.5 **↑**
*arsC1*	Arsenate reductase	AFM38847	2.9*↑	31.6 **↑**	2.7 **↑**
*arsC2*	Arsenate reductase	AFM38848	4.7*↑	36.4 **↑**	3.7 **↑**
*acr3*	Arsenic resistance protein	KDR86818	18.3 **↑**	23.9 **↑**	1.3
**Stress response**					
*katA*	Catalase-Peroxidase	KDR87137	2.4 **↑**	4.3 **↑**	9.9*↑
*sod1*	Superoxide dismutase	KDR88529	3.0 **↑**	3.0 **↑**	1.2
*sod2*	Superoxide dismutase	KDR91041	5.2 **↑**	7.0 **↑**	2.2 **↑**
*groEL*	Heat shock protein 60 family chaperone GroEL	KDR90204	2.4 **↑**	3.7 **↑**	5.7*↑
*ahpC*	Alkyl hydroperoxide reductase	KDR91031	2.3 **↑**	0.5 ↓	0.1* ↓
**Carbohydrate transport and metabolism**					
*pfp*	Pyrophosphate fructose 6-phosphate 1-phosphotransferase	KDR87902	2.8*↑	2.4 **↑**	2.2*↑
*gpmI*	2,3-biophosphoglycerate-independent phosphoglycerate mutase	KDR90307	3.6 **↑**	3.0 **↑**	2.7*↑
*galU*	UTP-glucose-1-phosphate uridylytransferase	KDR90225	0.7 ↓	0.1 ↓	0* ↓
*pckA*	Phosphoenolpyruvate carboxykinase	KDR87357	4.0 **↑**	2.7 **↑**	5.1*↑
*pdhB*	Pyruvate dehydrogenase E1 component beta subunit	KDR89058	2.7*↑	3.4 **↑**	4.3*↑
*acnA*	Aconitate hydratase	KDR88332	2.2 **↑**	2.9 **↑**	4.1 **↑**
*fumC*	Fumarate hydratase	KDR89425	2.7 **↑**	2.2 **↑**	5.3 **↑**
*sdhA*	Succinate dehydrogenase	KDR89039	2.7 **↑**	2.3 **↑**	2.7 **↑**
**Cell mobility**					
*mcp*	Methyl-accepting chemotaxis protein	AFM38850	2.7 **↑**	2.4 **↑**	1.6 **↑**
*fliC*	Flagella associated protein	KDR87546	1.6 **↑**	3.0 **↑**	2.2 **↑**
**Phosphonate and phosphinate metabolism and phosphate transportation**					
*phnI*	α-D-ribose 1-methylphosphonate 5-triphosphate synthase subunit	KDR86951	4.1*↑	1.7 **↑**	2.2*↑
*phnM*	α-D-ribose 1-methylphosphonate 5-triphosphate diphosphatase	KDR86941	2.8*↑	2.8 **↑**	1.6 **↑**
*pstS2*	Phosphate binding protein	KDR86346	2.0*↑	4.1 **↑**	2.4 **↑**
*ugpB1*	Periplasmic glycerol-3-phosphate-binding protein	KDR87393	5.3*↑	4.9 **↑**	4.4*↑
*ugpB2*	Periplasmic glycerol-3-phosphate-binding protein	KDR89469	8.7*↑	4.6 **↑**	18.7*↑
**Amino acid transport and metabolism**					
*glnA*	Glutamine synthetase	KDR90209	2.2 **↑**	1.8 **↑**	3.9*↑
*aspB*	Aspartate aminotransferase	KDR87822	2.7 **↑**	1.7 **↑**	2.7*↑
*lysC*	Aspartokinase	KDR87992	2.8 **↑**	1.7 **↑**	2.4*↑
*trpB*	Tryptophan synthase beta chain	KDR87480	3.9*↑	3.2 **↑**	2.0 **↑**
*dppA*	Dipeptide-binding ABC transporter	KDR88285	ND	ND	3.1*↑
*ddpA*	ABC transporter, substrate binding protein	KDR88329	ND	ND	0*↓
**Nucleotide metabolism**					
*surE*	5'-nucleotidase surE	KDR90749	ND	ND	0.2*↓
*cpdP*	3',5'-cyclic-nucleotide phosphodiesterase	KDR86320	25.9*↑	5.6 **↑**	6.1 **↑**
**Function unknown**					
AGRO_968	Hypothetical protein AGRO_968	KDR89367	ND	ND	4.6*↑
AGRO_2233	Hypothetical protein AGRO_2233	KDR87736	ND	ND	0.1*↓
AGRO_4594	Hypothetical protein AGRO_4594	KDR90647	ND	ND	2.3*↑

The proteomics data of strain GW4 with or without 50 μM Sb(III) are taken from Li et al., 2015.

* represents the proteomics data, the rests are qRT-PCR data. The data are present with the average of triplicate experiments.

### Transcription analysis in strains GW4, GW4-Δ*aioA* and GW4-Δ*aioA*-C with or without Sb(III)

**Fig 3:**
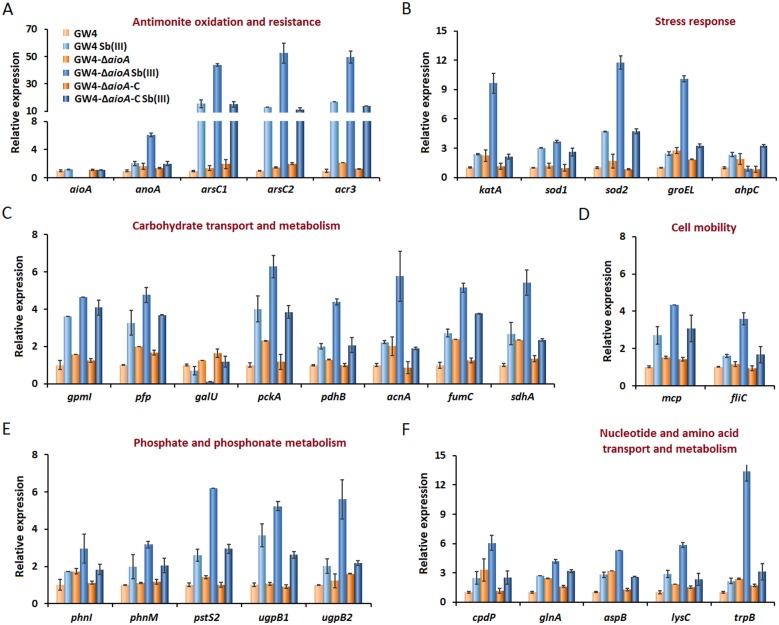
Quantitative reverse transcriptase-PCR analysis of the genes encoding proteins involved in antimonite oxidation and resistance (A), carbohydrate transport and metabolism (B), cell mobility (C), phosphate and phosphonate metabolism (D), and nucleotide and amino acid transport and metabolism (E) in *A*. *tumefaciens* strains. Total RNA was isolated from strain GW4, GW4-Δ*aioA* and GW4-Δ*aioA*-C cultured with or without 50 μM Sb(III) in CDM medium, respectively. Data are shown as the mean of three replicates, with the error bars representing ± SD.

Transcription analysis was performed by qRT-PCR in strains GW4, GW4-Δ*aioA* and GW4-Δ*aioA*-C with or without the addition of Sb(III) (Fig 3). The results indicated that the expression levels of the encoding genes were almost consistent with the proteomic data (Table 1 and Fig 2). As shown in Fig 3, the transcription of most of genes were induced by Sb(III) in strain GW4 except for *aioA* and the UTP-glucose-1-phosphate uridylytransferase gene *galU*. The deletion of *aioA* did not affect the transcription of genes involved in Sb(III) oxidation and resistance, cell mobility, and phosphate and phosphonate metabolisms but increased the transcription of genes involved in oxidative stress response, carbohydrate metabolism, nucleotide and amino acid metabolisms without Sb(III) (Fig 3). In GW4-Δ*aioA*, with Sb(III), the transcription of genes involved in all these metabolisms were increased, but the alkyl hydroperoxide reductase gene *ahpC* and *galU* were down-regulated (Fig 3).

### The cytoplasmic Sb(III) oxidase AnoA and Sb(III) resistant genes were up-regulated by Sb(III) and highly up-regulated in strain GW4-Δ*aioA*

The cytoplasmic Sb(III) oxidase *anoA* was significantly up-regulated by Sb(III) in strain GW4-Δ*aioA*, indicating that AnoA was more active to oxidize Sb(III) to Sb(V) inside the cells. This is in agreement with the higher Sb(III) oxidation efficiency as observed in strain GW4-Δ*aioA* (Fig 2B). The *ars* operon has been demonstrated to confer bacterial Sb(III) resistance, because the As(III) carrier protein ArsB is also responsible for Sb(III) efflux [7, 8]. However, genome analysis showed that the genome of strain GW4 contains an *ars* operon without *arsB*. Another trivalent metalloid/H^+^ antiporter gene *acr3* located in the arsenic gene island (contig 215) and cotranscribed with *arsC*, can substitute *arsB* for Sb(III) efflux with the hydrolysis of ATP [10]. The qRT-PCR analysis showed that the transcription levels of *arsC1*, *arsC2* and *acr3* were induced by Sb(III) in *A*. *tumefaciens* (Fig 3A). This is consistent with the observations in *E*. *coli* [33] and our previous study [16]. In addition, it has been reported that AioAB could catalyze Sb(III) oxidation *in vitro*, and the deletion of *aioA* significantly decreased Sb(III) resistance in strains GW4 and 5A [15], suggesting that Sb(III) might exhibit a higher toxicity in the absence of AioAB. Thus, the Sb(III) efflux and Sb(III) oxidation were increased in GW4-Δ*aioA* against the toxicity of Sb(III).

### The proteins associated with bacterial oxidative stress response were up-regulated by Sb(III) and highly up-regulated in strain GW4-Δ*aioA*

Antimony is known to interact with thiol groups of proteins and induce oxidative stress in cells [34]. Microorganisms have therefore developed defense mechanisms that either decrease the concentration of reactive oxygen species (ROS) or repair oxidative damage [35]. The superoxide (O_2_^•-^) generated from oxidative stress could be transformed to H_2_O_2_ in the presence of Sod, and the H_2_O_2_ is subsequently consumed by KatA [36]. In addition, the alkyl hydroperoxide reductase AhpC is also involved in the oxidative stress response. However, it has been reported that AhpC could only detoxify low concentrations of H_2_O_2_ [37, 38].

The increased transcription of *sod1*, *sod2*, *katA* and *ahpC* in strain GW4 in the presence of Sb(III) indicated that Sb(III) could induce bacterial oxidative stress response and these genes may act synergistically to decrease the concentration of ROS in the wild-type strain (Fig 3B). This is consistent with the observations in strain *Herminiimonas arsenicoxydans* in response to As(III) [39] and our recent observation that Sb(III) induced the cellular H_2_O_2_ content in strain GW4 [19]. The increased transcription of *katA* and *ahpC* in strain GW4-Δ*aioA* in the absence of Sb(III) suggested that the As(III)/Sb(III) oxidase AioAB may be associated with bacterial oxidative stress response. This is consistent with our recent observations that the deletion of *aioA* increased the cellular H_2_O_2_ content without Sb(III) [19]. However, In the presence of Sb(III), the cellular H_2_O_2_ content in the *aioA* mutant was significantly higher than that of wild-type strain [19]. Thus, the increased transcription of *sod1*, *sod2* and *katA*, and the decreased transcription of *ahpC* in strain GW4-Δ*aioA* hinted that the *aioA* mutant might be subjected to a higher toxicity caused by Sb(III) induced oxidative stress and the high concentration of cellular H_2_O_2_ was mainly scavenged by KatA rather than by AhpC.

To repair the oxidative damages, the general stress response was also induced by Sb(III) in *A*. *tumefaciens* strains. The heat shock protein GroESL, composed of GroES and GroEL, is one of the major heat shock chaperones in *E*. *coli*, which is essential for recovery of unfolded proteins and growth balance [40]. It has been shown that GroESL plays an important role in proteolysis and the degradation of proteins by ATP-dependent proteases [41]. The up-regulation of *groEL* in strain GW4 indicated that the heat shock response was also enriched to combat the toxicity of Sb(III) by refolding the misfolded proteins, which was coupled to energy consumption. The increased transcription of *groEL* in strain GW4-Δ*aioA* compared to the wild-type strain GW4 with or without Sb(III) suggested that the protein repair process was enhanced in the *aioA* mutant to tolerate the toxicity caused by oxidative stress.

### AioA affected carbon metabolism pathways

**Fig 4:**
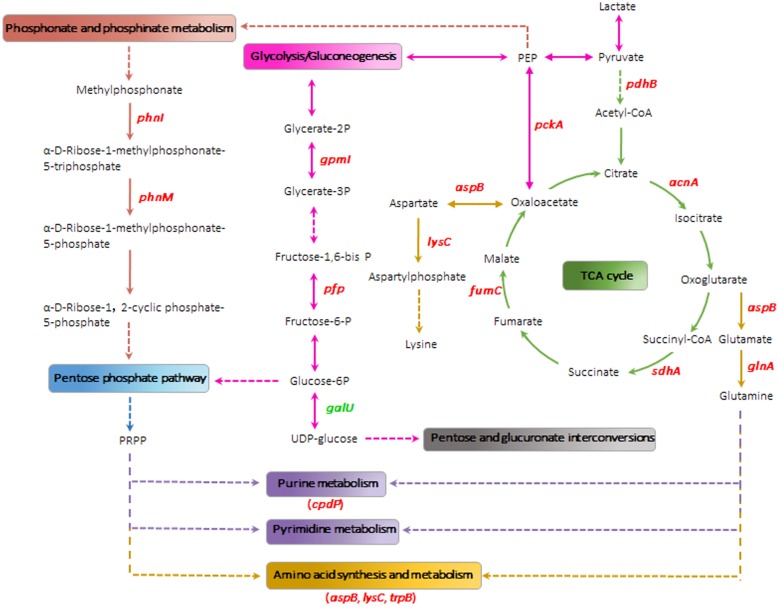
The metabolic pathways for carbohydrates, phosphonate and phosphinate, nucleotide and amino acids influenced by Sb(III) in *A*. *tumefaciens* GW4. Red text indicates the up-regulated genes, green text indicates the down-regulated genes. The dashed vector arrows indicate potential pathways that involve more than one intermediates and genes but none of which could be identified in the proteomic analysis. All reaction steps were derived from KEGG pathway maps.

**Fig 5:**
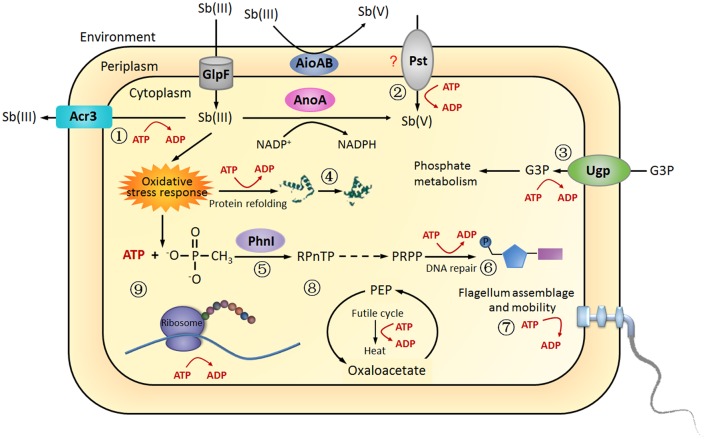
A proposed model of bacterial energy metabolism in response to Sb(III) in *A*. *tumefaciens* GW4. The energy generated from the process of Sb(III) oxidation was used to (1) Sb(III) efflux; (2) Pi or Sb(V) transportation; (3) G3P transportation; (4) protein refolding; (5) phosphonate metabolism; (6) DNA repair; (7) flagellum assemblage and mobility; (8) heat release and (9) protein synthesis. Such metabolisms were more activated with the disruption of *aioA*.

Based on the differentially expressed proteins and the cellular concentrations of ATP and NADH, the carbohydrate metabolic pathway in strain GW4 was analyzed. *A*. *tumefaciens* GW4 is a heterotrophic Sb(III)-oxidizing bacterium, which requires an organic compound as an energy source [17]. In this study, we used lactate as the carbon source and electron donor for promoting bacterial growth. The carbon metabolism of strain GW4 starts with lactate and subsequently flows into the TCA cycle and glycolytic/ gluconeogenic pathway (Fig 4). In the presence of PdhB, the pyruvate was catabolized to acetyl-CoA, which subsequently activated the TCA cycle (Fig 4). The increased transcription levels of *pdhB*, *acnA*, *fumC* and *sdhA* in the presence of Sb(III) indicated that the TCA cycle was induced by Sb(III) in strain GW4 (Fig 3C). Meanwhile, the up-regulation of PckA implied that the transformation between oxaloacetate and phosphoenolpyruvate (PEP) was increased to form an ATP-depleting futile cycle (Fig 5).

The up-regulation of GpmI and Pfp which transform glycerate-2P to glycerate-3P, and fructose-1,6P2 to fructose-6P, respectively, suggested that the PEP generated from oxaloacetate was transformed to glucose-6P through the gluconeogenic pathway (Fig 4). The glucose-6P was subsequently incorporated into the pentose phosphate pathway to generate phosphoribosyl pyrophosphate (PRPP), which is an intermediate produced from ribose-5-phosphate with the hydrolysis of ATP [40]. Conversely, glucose-6P could also be transformed to glucose-1P and could subsequently generate UDP-glucose in the presence of GalU (Fig 4). However, the transcription level of *galU* was down-regulated (Fig 3C), indicating that glucose-6P was prone to participate in the pentose phosphate pathway producing more PRPP rather than pentose and glucuronate interconversions (Fig 4).

The alternate carbon metabolic pathways induced by Sb(III) in strain GW4 were partially consistent with the cases of As(III). In heterotrophic As(III)-oxidizing strain *Herminiimonas arsenicoxydans* ULPAs1, proteins involved in the carbohydrate metabolism and TCA cycle were up-regulated in response to As(III) [42, 43]. The energy generated from the TCA cycle and As(III) oxidation was used for various metabolic activities against the toxicity of As(III) [42, 43]. Similarly, strain GW4 might also need a large amount of energy to combat the stress caused by Sb(III). In addition, the PRPP generated from the pentose phosphate pathway plays an important role in purine and pyrimidine nucleotide synthesis, salvage pathways and histidine metabolism [41]. Moreover, the pentose phosphate pathway is the first-line response to oxidative stress for the synthesis of nucleotides and NADPH to reduce DNA damage caused by ROS [44]. This was further supported by the increased expression of 3’,5’-cyclc-nucleotide phosphodiesterase CpdP, which is involved in purine synthesis and metabolism (Table 1 and Fig 3F). Thus, the up-regulation of the pentose phosphate pathway and the increased synthesis of PRPP in strain GW4 were associated with the repair of DNA damage in the presence of Sb(III) and these processes consumed part of the energy. The transcription levels of *gpmI*, *pfp*, *pckA*, *pdhB*, *acnA*, *fumC*, *sdhA* and *cpdP* were increased in strain GW4-Δ*aioA* without Sb(III) (Fig 3C), reflecting the oxidative stress caused by the deletion of *aioA*. Moreover, the further increased transcription of these genes and the decreased transcription of *galU* in strain GW4-Δ*aioA* with Sb(III) suggested that the *aioA* mutant might need more PRPP synthesis and energy generation for DNA and protein repair (Fig 5).

### The energy appeared to be partially used for bacterial mobility and released in the form of heat

Similar to arsenic, it is important to clarify the bacterial chemotaxis towards Sb(III) to further understand the global response to this metalloid. The genome of strain GW4 contains a single-copy gene *fliC*, which encodes the polymerized subunits (KDR87546) of the bacterial flagellum and is involved in bacterial motility. In addition, a chemotaxis protein encoding gene *mcp* is located adjacent to the *aio* operon in the arsenic gene island (contig 215, AFM38850) and is responsible for As(III) chemotaxis, and the GW4-Δ*aioA* showed null As(III) oxidation and no chemotaxis towards As(III) [45]. The increased transcription levels of *mcp* and *fliC* in strains GW4 and GW4-Δ*aioA* indicated that bacterial mobility was enhanced in response to Sb(III) (Fig 3D), which was consistent with the phenotype of bacterial mobility towards Sb(III).

*A*. *tumefaciens* GW4 is a heterotrophic As(III)/Sb(III)-oxidizing strain with a high Sb(III) tolerance (8 mM). Even though the Sb(III) did not obviously enhance the growth of strain GW4 as reported for As(III) oxidation [22], low amounts of Sb(III) (e.g. 10, 25, 50 μM) appeared not so toxic to strain GW4, instead, the amount of ATP and NADH were increase (Fig 1E and 1F), indicating that Sb(III)-oxidation may also produce energy as in the case of As(III) oxidation. Thus, the cells showed positive swarm to Sb(III). However, with high amounts of Sb(III) (e.g. 200, 500 μM), the growth of GW4 was inhibited and the strain would swarm away from it due to the high toxicity of Sb(III) (data not shown). In addition, even though the deletion of the periplasmic *aioA* resulted in more Sb(III) to inside cells, the cytoplasmic Sb(III) oxidation was increased and the carbon metabolism was activated. The enhanced Sb(III) oxidation and carbon metabolism in GW4-Δ*aioA* is correlated with the increased amounts of ATP and NADH (Fig 1E and 1F). These results are similar with the observations in the As(III)-oxidizing strains *A*. *tumefaciens* GW4 and *H*. *arsenicoxydans* ULPAs1, which exhibited positive chemotaxis towards As(III) [21, 42]. It is known that the assembly of flagella and the function of Na^+^-driven flagellar motor activity was coupled to energy consumption [46], and thus, the energy was partially used for bacterial mobility (Fig 5).

The heat produced by bacteria is tightly coupled to bacterial growth and metabolic reactions. However, some bacteria can consume higher amounts of energy through futile cycles without concomitant biomass production and the energy sources are converted to heat [47]. In this study, the PckA was up-regulated in the presence of Sb(III) (Fig 3C), indicating that the PckA catalyzed PEP-oxaloacetate futile cycle consumed part of the energy in *A*. *tumefaciens* strains and the energy-spilling reaction was enhanced in GW4-Δ*aioA*. These results suggested that the energy generated from the process of Sb(III) oxidation was partially released in the form of heat, and the *aioA* mutant strain might be resistant to Sb(III) by the increased release of heat (Fig 5).

### Phosphonate and phosphinate metabolism and phosphate transportation are in response to Sb(III) oxidation

Using a combination of proteomics and qRT-PCR analysis, we found that the transcription of genes involved in phosphonate and phosphinate metabolism (*phnI*, *phnM*, *pstS2*, *ugpB1* and *ugpB2*) was induced by Sb(III) in strain GW4 and GW4-Δ*aioA* (Table 1 and Fig 3E). The PEP generated from the glycolytic pathway could be transformed to methylphosphorate and ultimately enter the pentose phosphate pathway under the function of the *phn* gene cluster (Fig 4). The enhanced phosphonate and phosphinate metabolisms increased the production of PRPP along with the gluconeogenic pathway (Fig 4), suggesting that the heterotrophic Sb(III)-oxidizing strain required more DNA repair and amino acids synthesis processes in response to Sb(III).

In addition, the transformation of methylphosphorate to phosphate and methane could also be a resource of Pi under phosphate starvation conditions [48]. The CDM medium used in this study contained ~53 μM Pi, which is a low-Pi condition for bacterial growth. The hydrolytic cleavage of the C-P bond was relayed on the proteins encoding by the *phn* operon and coupled to the hydrolyzation of ATP [48]. Thus, it is rational to assume that the *A*. *tumefaciens* strains require more phosphate for bacterial growth and Sb(III) resistance. This was further supported by the up-regulation of UgpB1, UgpB2 and PstS2 (Table 1 and Fig 3E), which belong to the high-affinity Pi transport system encoded by the *ugp* and *pst* operon, respectively [49, 50].

In the case of As(III), phosphate transport proteins were also inducible by As(III) in the As(III)-oxidizing strain *H*. *arsenicoxydans* ULPAs1, *Comamonas* sp. CNB-1 and *A*. *tumefaciens* 5A [43, 51]. Based on the molecular similarities between Pi and As(V), it has been verified that the inorganic phosphate carriers were responsible for As(V) transportation in both prokaryotes and eukaryotes [22]. However, whether the phosphate transport system Pst could transport Sb(V) into the cells remains open for further study. In this study, the increased phosphonate and phosphinate metabolism and phosphate transportation in *A*. *tumefaciens* strains suggested that Sb(III) interfered with DNA and protein synthesis and the *aioA* mutant required more PRPP and Pi for complementary synthesis and repair of biological macromolecules in response to Sb(III).

### Proteins involved in amino acid synthesis and metabolism are up-regulated by Sb(III) and highly up-regulated in strain GW4-Δ*aioA*

The transcription levels of *glnA*, *aspB*, *lysC* and *trpB* were also found to be induced by Sb(III) in wild-type strain GW4 and the *aioA* mutant (Fig 3F). KEGG pathway analysis showed that AspB and LysC are responsible for the synthesis of lysine from oxaloacetate, an intermediate of the TCA cycle. The oxaloacetate was transformed to aspartate through transamination catalyzed by AspB, proceeding on to the production of aspartylphosphate in the presence of LysC (Fig 4). In addition, AspB could also catalyze oxoglutarate, another intermediate of the TCA cycle, to glutamate and subsequently to glutamine, which participates in purine, pyrimidine and amino acid metabolism (Fig 4).

KEGG pathway analysis also showed that GlnA, AspB, LysC and TrpB are involved in the synthesis and metabolism of various amino acids, such as Arg, Ala, Asp, Glu, Gly, Lys and Pro, although the key metabolism pathways of these amino acids were not identified in this study. The up-regulation of these proteins is in agreement with previous studies showing that the proteins involved in transcription and translation were increased in As(III)-oxidizing strain *H*. *arsenicoxydans* ULPAs1 and *Comamonas* sp. CNB-1 in response to As(III) [41, 49]. Furthermore, the studies on drug resistance of *Leishmania* showed that amino acid synthesis and metabolism were increased in most of the Sb(III)-resistance *Leishmania* [52–54]. In this study, the deletion of *aioA* increased the genes involved in amino acid synthesis and metabolism compared with strain GW4 without Sb(III) because the oxidative stress also contributed to the damage of protein. In the presence of Sb(III), amino acid synthesis and metabolism were increased in strain GW4 for protein complementary synthesis and these processes were more active in the *aioA* mutant due to the higher toxicity of Sb(III) without AioA. Moreover, the amino acid synthesis and metabolism also consumed large amounts of energy generated from the process of Sb(III) oxidation (Fig 5).

## Conclusion

The observations of this study revealed that Sb(III)-oxidation may also produce energy as in the case of As(III) oxidation in *A*. *tumefaciens* GW4. The AioAB is responsible for Sb(III) oxidation in the periplasm, without AioAB, more Sb(III) would enter the cells, thus, the cytoplasmic Sb(III) oxidase AnoA and proteins involved in cellular oxidative stress response were significantly up-regulated and the Sb(III) oxidation efficiency was increased. In addition, carbon metabolism, amino acid synthesis, DNA repair, bacterial mobility and heat release were all enhanced in response to Sb(III).

## Supporting information

S1 Table Primers used in the quantitative RT-PCR analysis. (PDF)
