# Supplementary material for: Effects upon metabolic pathways and energy production by Sb(III) and As(III)/Sb(III)-oxidase gene aioA in Agrobacterium tumefaciens GW4
Source: PLoS One. 2017 Feb 27;12(2):e0172823. doi: 10.1371/journal.pone.0172823 (PMC5328403; doi:10.1371/journal.pone.0172823)
Supplement: S1 Table — (PDF) [file pone.0172823.s001.pdf]

Effects upon Metabolic Pathways and Energy Production by Sb(III) and As(III)/Sb(III)-oxidase Gene *aioA* in *Agrobacterium tumefaciens* GW4

Jingxin Li, Birong Yang, Manman Shi, Kai Yuan, Wei Guo, Mingshun Li, and Gejiao Wang*

S1 Table. Primers used in the quantitative RT-PCR analysis.

Primer pair	Primer sequence
P16S-F/R	5'GGTATGGGCATTGGAGACGA3' / 5'GGCAACTAAGGGCGAGGG3'
PaioA-F/R	5'CAGTATGTGCTGCGTGTC3' / 5'GAGGCGGCCGTAGAT3'
PanoA-F/R	5'TCATGGTCGAAAGCATCGG3' / 5'GCGGTTCTGGACGATGTCATA3'
ParsC1-F/R	5'TTCCCCACGCGAATTACTTC3' / 5'TCCATCATAGCATCGACAAGGT3'
ParsC2-F/R	5'GCCGGTTTGTCGCCTATTC3' / 5'AAGCCGGTCGACGGATAAC3'
Pacr3-F/R	5'GTTCCTCGCGGGCTATCTG3' / 5'CAGCGTGATCGGGCTGAT3'
PkatA-F/R	5'CGGCCTCGGGTAAATCG3' / 5'CGGCCTTCAATGCTTCGA3'
Psod1-F/R	5'TAAGCAGAATTTCGCCCTCT3' / 5'ACATCGCAGCCAAGAAGC3'
Psod2-F/R	5'GACAAGTTCAAGGCCGATTT3' / 5'CGTAGTCCCAGTTGATGAGG3'
PgroEL-F/R	5'CACCAAGGACGGCGTTTC3' / 5'CTTGAGGTCCATCGGGTTC3'
PahpC-F/R	5'TTTCGGCGGCAAGAAGGT3' / 5'CGGCGAGGAAGTGGATTTT3'
Ppfp-F/R	5'GCAGTTCGCCAGCCTCAT3' / 5'TTTCCGCCCTTGATACGC3'
PgpmI-F/R	5'GTGGATCGTGGGCTTGGT3'/ 5'CAGCGGCACATTCATCAGTT3'
PgalU-F/R	5'TAAGCAGAATTTCGCCCTCT3' / 5'GCCTGCCAGTTCACCAGA3'
PpckA-F/R	5'CTTGATTGCCTTGGCGTAGC3' / 5'TGTCGGTCCTGAGGGTGATT3'
PpdhB-F/R	5'GCGGCCTATCGTCGAATTC3'/ 5'CTGGCCACCCGACATGTAA3'
PacnA-F/R	5'ACCACTCGGTCATCGTCG3' / 5'ATCGTCTCGCCATCCTCTT3'
PfumC-F/R	5'AAAATTCGCTGATGCTGGTG3' / 5'TCCGGGCGTACAAACTGG3'
PsdhA-F/R	5'ACCATCCATCGCTTTGCC3' / 5'CGGTCGGGTGGAACTGAA'
Pmcp-F/R	5'TCGCAGCCACAACCAAA3' / 5'CCGATACCCGCAAGATAGA3'
PfliC-F/R	5'CGCTTTCCTCGGTTTC3' / 5'CGACGCCTTCTTCAGTT3'
PphnI-F/R	5'CGTTGAGACAGCCACCCAG3' / 5'CCAGAAGGAAACCCTCGTCA3'
PphnM-F/R	5'CGGCGTGATCTGGGACAA3'/ 5' CACATCCGAGGAAGCGACTT3'
PpstS2-F/R	5'TTTCGGCCTCGCTTTCTATG3' / 5'ACGCCCAGATGAGCCTTCTT3'
PugpB1-F/R	5'GGCGTGCGTTATTTCTATGCT3' / 5'CGCAACCTGCTCGAACTCTT3'
PugpB2-F/R	5'GGCTACATCCCGGTTACCAA 3'/ 5'GGCTTTCGATGGCCTTTTC3'
PglnA-F/R	5'GGTTCTGCTCGCCTACTCCG3' / 5'GCGGGAAGGTCGTAAAGGT3'
PaspB-F/R	5'CACCATCGCCGTTACCCA3' / 5'GCTTGTAGTCCAGGCCGTTTT3'
PlysC-F/R	5'AGGAAATGCTCGAAATGGC3' / 5'TCATCCTCGTCACAAATCAAAG3'
PtrpB-F/R	5'GAACTGGCGGATTACGACG3' / 5'ATGGTGCAGGAAGGTGGG3'
PcpdP-F/R	5'GCCATGAGGCCGATGTGA3' / 5'CGGAACTGTAGGCGATGC3'
